# Role of fibular autograft in ankle arthrodesis fixed using cannulated screws: a proportional meta-analysis and systematic review

**DOI:** 10.1038/s41598-023-46034-x

**Published:** 2023-10-30

**Authors:** Alessio Bernasconi, Antonio Izzo, Martina D’Agostino, Massimo Mariconda, Antonio Coviello

**Affiliations:** 1 Department of Public Health, Trauma and Orthopaedics, University of Naples Federico II, Naples, Italy; 2 Department of Neurosciences, Reproductive and Odontostomatological Sciences, University of Naples Federico II, Naples, Italy

**Keywords:** Outcomes research, Cartilage, Skeleton, Bone, Trauma

## Abstract

Ankle arthrodesis is commonly performed to treat end-stage ankle osteoarthritis. The aim of this study was to determine whether the use of fibular autograft might increase the fusion rate and decrease the complication rate in ankle arthrodesis (AA) fixed using cannulated screws. To perform this PRISMA-compliant proportional meta-analysis, multiple databases were searched for studies in which patients undergone AA (using exclusively cannulated screws and augmented with fibular bone graft) were followed. The characteristics of the cohort, the study design, surgical details, the nonunion and complication rate at the longest follow-up were extracted and recorded. The modified Coleman Methodology Score (mCMS) was applied to appraise the quality of studies. Two groups were built: arthrodeses fixed with screws combined with cancellous autograft (G1) and arthrodeses fixed with screws combined with cancellous autograft and augmented with a lateral fibular onlay (G2). A third group (arthrodeses fixed with screws and no graft, G3) was extracted from previous literature for a further comparison. Overall, we included 306 ankles (296 patients) from ten series (ten studies). In G1 and G2 there were 118 ankles (111 patients) and 188 ankles (185 patients), respectively. In patients where cancellous autograft was used, a further augmentation with a fibular lateral strut autograft did not change significantly the nonunion (4% [95% CI 1–9] in G1 vs. 2% [95% CI 0–5) in G2, *p* = 0.99) nor the complication rate (18% [95% CI 0–36] in G1 vs. 13% [95% CI 6–21) in G2, *p* = 0.71). Upon comparison with 667 ankles (659 patients, G3) in which arthrodeses had been performed without grafting, the nonunion and complication rates did not differ significantly either (pooled estimates: 3% [95% CI 1–3) in G1 + G2 vs. 3% [95% CI 2–4] in G3, *p* = 0.73 for nonunion; 15% [8–23] in G1 + G2 vs. 13% [95% CI 9–17] in G3, *p* = 0.93 for complications). In ankle arthrodesis fixed with cannulated screws combined with cancellous autograft at the fusion site, a construct augmentation with a distal fibular onlay strut graft positioned laterally at the ankle joint does not reduce the risk of nonunion or complication. In general, the use of bone graft does not influence significantly the nonunion nor the complication rate as compared to non-grafted screw-fixed ankle arthrodeses.Kindly check and confirm the corresponding author mail id is correctly identified.It's all correct

## Introduction

Severe ankle osteoarthritis resistant to non-surgical treatments represents a common indication for ankle arthrodesis (AA) or Ankle Arthroplasty^1–4^. Multiple studies have been undertaken over the last years comparing these two techniques, often concluding that both procedures could lead to a satisfactory outcome in this setting. There is good consensus about the fact that for ankles presenting after an infection, or with marked joint deformity or with bony defects AA represent the gold standard. From a technical point of view, AA can be performed using a number of fixation methods (such as cannulated screws, one or more plates, a nail or an external fixator) and following various strategies (arthroscopically-assisted procedures, use or not of bone or synthetic graft, one or two-stage procedures) depending on the specific case^3, 5–10^. Surgeons usually rely on their experience and on the patient’s conditions in order to choose a fixation method to achieve the fusion of the ankle joint, since no technique has been proven more effective than another^11, 12^.

Some recent literature has been produced focusing on AA performed using cannulated screws^8, 13–15^, overall suggesting that using different number of screws or combining various thread designs or positioning metalwork in different configurations does not clearly affect the success rate of the procedure. At times, surgeons may decide to place some cancellous bone at the fusion site or to use an onlay fibular strut autograft placed laterally as a bridge between the tibia and the talus, with the aim to add both a biological and mechanical augmentation to the fixation^16^. A systematic review (without a formal meta-analysis) performed by Heifner et al. has included studies dealing with open ankle arthrodeses fixated using screws published until October 2019. The authors have concluded that using bone graft did not have a significant effect on union rates^16^. However, to the best of our knowledge, a consensus about the value of fibular bone grafting both for open or arthroscopically-assisted procedures is still lacking.

In this setting, we performed a proportional meta-analysis to determine whether the use of a distal fibular autograft (as cancellous bone interposed at the fusion site or as lateral strut) in AA fixed using cannulated screws might influence the union and complication rate. We hypothesized that, in ankle arthrodesis fixed with cannulated screws combined with cancellous autograft at the fusion site, augmenting the fixation with a distal fibular onlay strut graft positioned laterally at the ankle joint might reduce the risk of nonunion or complication. A second hypothesis was that the use of bone graft at the fusion site might increase the union rate and reduce the number of complications as compared to non-grafted constructs.

## Results

### Study population

**Figure 1:**
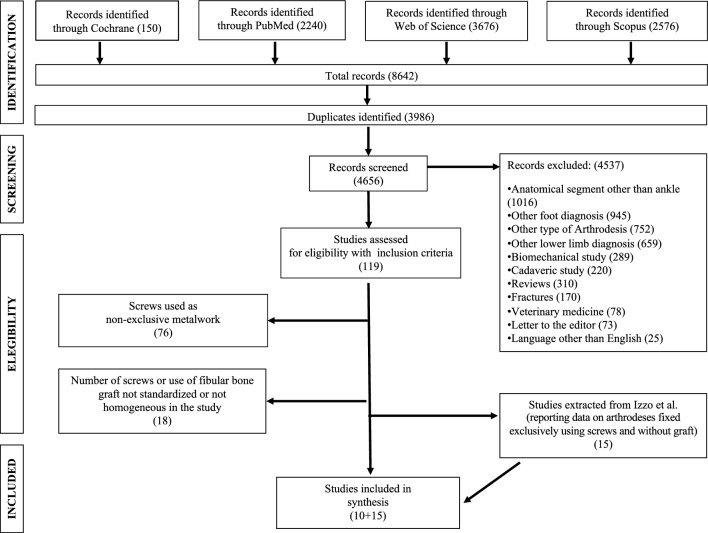
Flow chart for studies included in this systematic review.

Overall, we included 306 ankles (296 patients) from ten series (ten studies) (Fig. 1)^17–26^.

In G1 and G2 there were 118 ankles (111 patients) and 188 ankles (185 patients), respectively (Table 1). Raw data of a third group (G3, 15 studies) composed of 667 ankles (659 patients, G3) in which arthrodeses had been performed without grafting, was extracted from the study by Izzo et al.^13^ (Fig. 1) (Table 1).

**Table 1:** Main characteristics of studies included in this review.

Author (year)	Study design	LoE	Surgical access	Arthroscopic procedure (*Y/N*)	mCMS
Screws + Cancellous graft (G1)					
Chen (1996)	Retr—Non Comp	IV	Lateral	No	43
Nielsen (2008)	Retr—Comp	III	Lateral	No	56
Smith (2013)	Retr—Comp	IV	Lateral	No	46
Kim (2020)	Retr—Comp	III	Lateral	No	40
Screws + Cancellous graft + Lateral Fibular Autograft (G2)					
Monroe (1999)	Retr—Non Comp	IV	Leteral	No	41
Kennedy (2006)	Retr—Non Comp	IV	Lateral	No	60
Colman (2007)	Retr—Non Comp	IV	Lateral	No	40
Akra (2010)	Retr—Non Comp	IV	Lateral	No	44
Napiontek (2015)	Retr—Non Comp	IV	Lateral	No	42
Lee (2020)	Retr—Non Comp	IV	Lateral	No	39
Only screws (no autograft) (G3)*					
Sward (1992)	Retr—Non Comp	IV	Posterior	No	48
Stranks (2004)	Retr—Non Comp	IV	Anterior	No	42
Winson (2005)	Retr—Non Comp	IV	–	Yes	60
Ferkel (2005)	Retr—Non Comp	IV	–	Yes	46
Nielsen (2008)	Retr—Comp	III	–	Yes	56
Odutola (2011)	Retr—Non Comp	IV	–	Yes	35
Dannawi (2011)	Retr—Comp	IV	–	Yes	58
Shuh (2011)	Retr—Non Comp	IV	Anterior	No	43
Hendrickx (2011)	Retr—Non Comp	IV	Medial + Lateral	No	56
Duan (2016)	Retr—Non Comp	IV	–	Yes	61
Vaishya (2017)	Retr—Non Comp	IV	–	Yes	44
Kolodziej (2017)	Retr—Non Comp	IV	–	Yes	39
Yang (2020)	Retr—Comp	III	–	Yes	65
Teramoto (2020)	Retr—Comp	III	–	Yes	43
Fisher (2021)	Retr—Comp	III	Anterior	No	66

Prosp, Prospective; Retr, Retrospective; Comp, Comparative; Non Comp, Non Comparative; LoE, Level of Evidence; mCMS, modified Coleman Methodology Score.

*Data extracted from Izzo et al.

In the three groups, the sample size (*p* = 0.52), mean age of patients (*p* = 0.33), sex distribution (*p* = 0.67) and follow-up (*p* = 0.27) were comparable (Table 2). The mean mCMS (48.8 ± 9, range, 35–66) revealed only an overall moderate quality of studies, with a risk of bias which was comparable in the three groups (46.2 in G1, 45.6 in G2 and 50.8 in G3; *p* = 0.43).

**Table 2:** Baseline characteristics of the cohorts investigated in the studies included in this review.

	Screws + cancellous graft (G1)		Screws + cancellous graft + lateral fibular autograft (G2)		Only screws (G3) *		*p-*value
	Mean ± SD	Range	Mean ± SD	Range	Mean SD	Range	
Ankle *(N)*	29.5 ± 18.7	12–49	44.4 ± 28.4	19–118	31.3 ± 9.9	23–48	0.52
Patients* (N)*	27.7 ± 18	12–48	43.9 ± 28.3	18–116	30.8 ± 10	22–48	0.48
Age (*y)*	52.2 ± 3.2	48–56	56.8 ± 7.4	43.7–70	52.6 ± 5.7	46–62	0.33
Sex *(%F)*	0.39 ± 0.08	0.3–0.5	0.41 ± 0.16	0–0.6	0.30 ± 0.25	0–0.6	0.67
Follow-up *(m)*	25.8 ± 16.2	12–48	47.9 ± 26	12–110	41.6 ± 30.9	15–102	0.27

SD, Standard deviation; N, Number; F, Female; y, Years; m, Months.

*Data extracted from Izzo et al.

### Cancellous bone versus cancellous bone + fibular onlay graft

**Figure 2:**
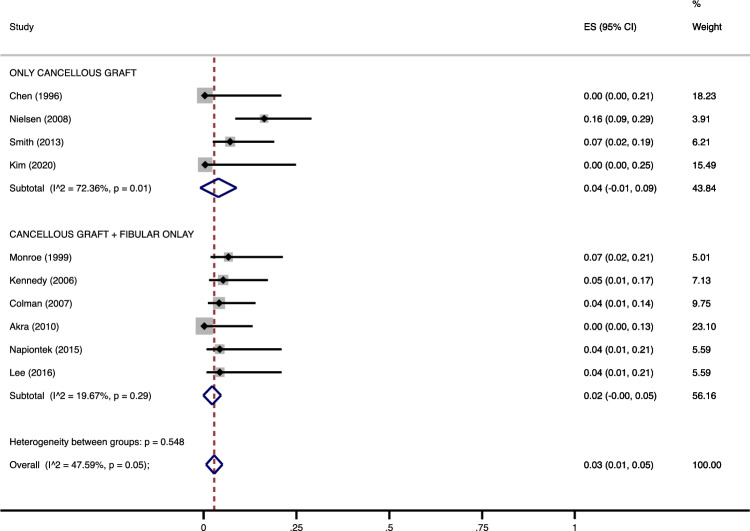
Meta-analysis of the proportion of nonunions in patients undergone Ankle Arthrodesis fixed with cannulated screws in which fibular cancellous graft was used alone or in combination with a lateral distal fibula strut graft. Output generated by the Stata procedure *metaprop*.

**Figure 3:**
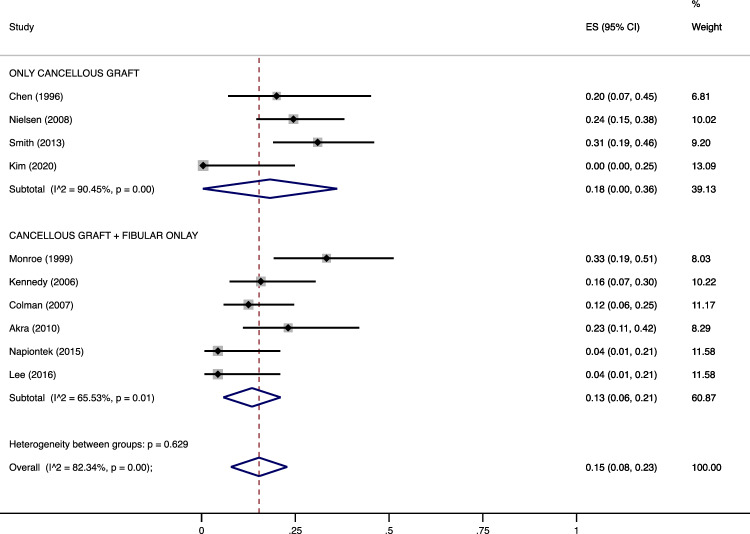
Meta-analysis of the proportion of complications in patients undergone Ankle Arthrodesis fixed with cannulated screws in which fibular cancellous graft was used alone or in combination with a lateral distal fibula strut graft. Output generated by the Stata procedure *metaprop*.

In patients where cancellous autograft was used, a further augmentation with a fibular lateral strut autograft did not change significantly the nonunion (4% [95% CI 1–9] in G1 vs. 2% [95% CI 0–5) in G2, *p* = 0.99) (Fig. 2) nor the complication rate (18% [95% CI 0–36] in G1 vs. 13% [95% CI 6–21) in G2, *p* = 0.71) (Fig. 3). For the nonunion rate, intra-group heterogeneity was significant in G1 (*p* = 0.01) but not in G2 (*p* = 0.29), while inter-group heterogeneity was nonsignificant (*p* = 0.548), supporting the pooling of data into one pooled measure (3%, [95% CI 1–5]) (Fig. 2). For the complication rate, intra-group heterogeneity was significant both in G1 and G2 (*p* < 0.001 and *p* = 0.01, respectively), but intergroup heterogeneity was nonsignificant (*p* = 0.629) with a pooled measure at 15% [95% CI 8–23] (Fig. 3).

In the manuscript we found that reference [8,17,25,28] is a duplicate of [16,18,31,33] and hence the repeated version has been deleted. Please check.Apologies for the mistake, thank you.

### Autograft versus no autograft

**Figure 4:**
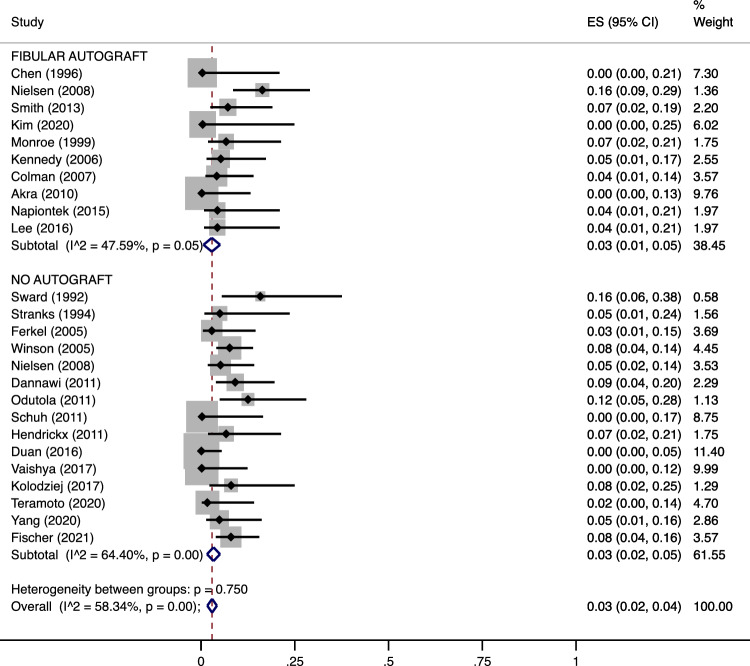
Meta-analysis of the proportion of nonunions in patients undergone Ankle Arthrodesis fixed with cannulated screws and augmented with a fibular autograft (including both only cancellous graft and cancellous graft augmented with a lateral fibular strut graft) versus Arthrodesis performed without autograft. Output generated by the Stata procedure *metaprop*.

**Figure 5:**
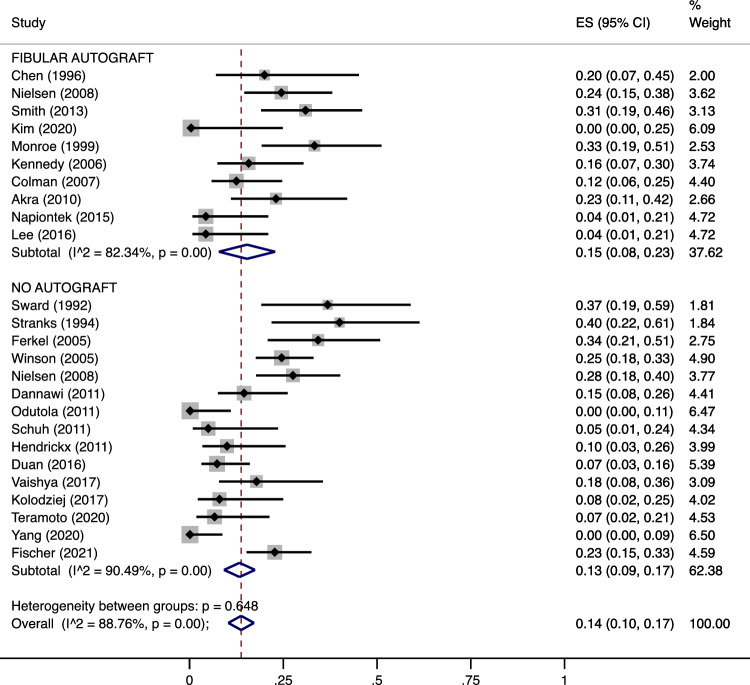
Meta-analysis of the proportion of complications in patients undergone Ankle Arthrodesis fixed with cannulated screws and augmented with a fibular autograft (including both only cancellous graft and cancellous graft augmented with a lateral fibular strut graft) versus Arthrodesis performed without autograft. Output generated by the Stata procedure *metaprop*.

Upon comparison between AA performed with autograft and without graft, the nonunion and complication rates did not differ significantly either (pooled estimates: 3% [95% CI 1–3) in G1 + G2 vs. 3% [95% CI 2–4] in G3, *p* = 0.73 for nonunions (Fig. 4); 15% [8–23] in G1 + G2 vs. 13% [95% CI 9–17] in G3, *p* = 0.93 for complications (Fig. 5)). For what concerns the nonunion rate, intragroup heterogeneity was nonsignificant in G1 + G2 (*p* = 0.05) and significant in G3 (*p* < 0.001). However, the intergroup heterogeneity was nonsignificant (*p* = 0.750) with a pooled estimate at 3% [95% CI 2–4) (Fig. 4). Regarding the complication rate, the intragroup heterogeneity was significant both in G1 + G2 and in G3 (*p* < 0.001 for both), while there was no significant intergroup heterogeneity (*p* = 0.648) with a pooled estimate at 14% [95% CI 10–17) (Fig. 5).

## Discussion

The main finding of this study is that in ankle arthrodeses fixed using cannulated screws the use of fibular bone graft, whether as standalone cancellous bone interposed at the tibiotalar fusion site or as cancellous bone augmented with an onlay lateral distal fibular graft, does not significantly influence the union or the complication rate. In fact, the risk of nonunion did not differ between grafted constructs and only-screws fixation methods.

With regard to the fibular bone graft, our findings support the idea that it may be not necessary in the majority of AAs, which corroborates some previous literature. In a recent systematic review, Heifner et al. reported a similar fusion rate in AA both with and without bone graft and fibular onlay (94.7% and 95.3%, respectively), concluding that the graft was not needed routinary in AA and should be dedicated to patients at high-risk of failure^16^. Interestingly, in their analysis the authors used the CMS to assess the quality of studies included which scored at 60, being not far from our 48.8 and confirming the only moderate quality of literature produced in this field^16^. On the other side, it should be emphasized that, as compared to our study, Heifner et al. adopted slightly different inclusion criteria since they excluded studies reporting arthroscopic arthrodeses (which is nowadays commonly performed in association with screw fixation) and including studies with a follow-up shorter than 12 months (which limits the strength of their conclusion in terms of complication rate). In another study by Duan et al. the use of two cannulated screws in arthroscopic AA led to a fusion rate of 100% with no use of bone graft^27^. Regardless of the use of fibular graft, in our study the pooled fusion rate was at least 96% (with a pooled nonunion rate ranging from 2 to 4%), which could be retained as indicative value for arthrodeses fixed using cannulated screws. It should be noted, however, that we included both open and arthroscopic AAs in the same group.

From a technical standpoint, the use of a fibular onlay autograft inevitably requires an open procedure which should inherently carry a higher risk of complications as compared to arthroscopic arthrodeses^28^. Also, preparing the distal fibula before using it as a graft (which includes harvesting it, shaping and/or morselising it) may require additional surgical time in those settings where a limited number of scrubbed assistants are available during surgery. While the complication rate in the studies selected in our analysis varied quite largely, ranging from 0 to 30%^18, 19, 21–24, 26, 29, 30^, the pooled estimate for complications in arthrodeses where fibular graft was used was 15%, being slightly higher but not statistically different from the complication rate of only-screws constructs (13%). Considering the greater number of screws used to stabilise a lateral fibular strut, we would have expected a higher complication rate related to irritation from metalwork in this group, which was not the case. Also, it should be emphasized that unfortunately data coming from a number of high-quality studies, such as the longitudinal cohort study recently published by Abuhantash et al. in which 351 AAs were followed up at 5 years, could not be used in this meta-analysis due to the heterogeneity of fixation methods (i.e., cross-screws, retrograde nailing, or plate fixation) reported in their study^28^. Anyway, along with the findings related to nonunion, we believe that the numbers related to the risk of complication might be extremely valuable in the clinical setting to correctly inform the patient during the pre-surgical counselling for these procedures.Please confirm the section headings are correctly identified.They are correct

This study is not without limitations. First, the Level of Evidence provided in this meta-analysis is IV since most studies included are of Level III and IV studies, which weakens the strength of our conclusions. Second, no clinical data has been reported in our analysis. Although this would have undoubtedly been useful, in primary studies we did not find sufficient clinical raw data to run any meaningful statistical analysis on this. Third, the comparison with only-screw constructs was performed using historical data taken from another study by Izzo et al., which inherently increases the risk of bias for our findings. However, since data extracted from that study were almost identical to the current work, we reckon that the results of the comparison performed in this meta-analysis might be still reliable and meaningful for clinicians. Fourth, intragroup heterogeneity was significant for most of the groups analyzed in this study. Although this may not be strictly interpreted as lack of consistency between studies in the same group, it should be taken into account when analysing our findings. In our opinion this statistical heterogeneity likely corresponds to differences in study design and surgical technique of primary studies. Even if AA generally represents the gold standard to treat ankle osteoarthritis while fibula autograft is usually considered in severe bone loss or deformity, the procedure can be carried out in multiple ways according to various habits for surgeons around the world. On the other side, intergroup assessments always revealed a good consistency among studies, which represents a further strength of our study. Finally, we acknowledge that other factors that have a known influence on bone healing and postoperative complications in AA (such as diabetes, body mass index and smoking) have not been evaluated in this study.

In conclusion, this proportional meta-analysis suggests that in ankle arthrodesis fixed with cannulated screws combined with cancellous autograft at the fusion site, a construct augmentation with a distal fibular onlay strut graft positioned laterally at the ankle joint does not reduce the risk of nonunion or complication. In general, the use of bone graft does not influence significantly the nonunion nor the complication rate as compared to non-grafted screw-fixed ankle arthrodeses. The quality of evidence provided in literature so far is only moderate, therefore high-level comparative and prospective studies around the use of graft in ankle arthrodesis are warranted.

## Methods

### Protocol and registration

This systematic review was designed according to the Preferred Reporting Items for Systematic reviews and Meta-Analyses (PRISMA). It was prospectively registered as a part of larger project on AA in the PROSPERO database (CRD42022322784).

### Eligibility criteria

The inclusion criteria were as follows: studies reporting data after AA (open or arthroscopically-assisted) stabilized using only screws in patients aged between 15 and 85 years; clear description of the surgical technique with one or more statements about the number of screws used and a systematic use of two or more screws and 1) a distal fibula autograft used as cancellous bone interposed between the tibia and the talus or 2) a distal fibula autograft used as cancellous bone at the fusion site augmented with a structural lateral construct or) in the whole cohort; studies including a sample size larger than 10 ankles; assessment of radiographic results through pre- and post-operative weightbearing standardized radiographs; reporting the fusion rate and complications after AA; minimum follow-up of 12 months; clinical studies; published in any language; full text availability either online either after direct contact with the authors.

Exclusion criteria were: studies reporting results after AA stabilized using other methods (nail, external fixator, plate, hybrid constructs); studies in which screws were used as unique way of fixation but without clear definition about the use of bone graft; data on skeletally-immature patients; case reports, biomechanical studies, cadaveric studies, expert opinions, letters to the editor, studies on animals and instructional courses. Narrative or systematic reviews were also excluded from this study but references were double checked in order to identify potential eligible studies.

### Information sources and search

A systematic search was conducted on Pubmed, Embase, Cochrane Library and Scopus, from the earliest entries through April 30, 2023 with the following key words and Boolean operators: ((ankle) AND (arthrodes*)) OR ((ankle) AND (fusion)). Additional studies were identified in the bibliographies of articles. Two reviewers (AI and MD) independently screened the results of the research, then the full text of eligible studies was analyzed. Disputes were resolved by the senior author (AB). Unpublished studies and gray literature were not considered.

### Data charting and items

Data were charted independently by two investigators (AI and MD) using an Excel sheet. Data were harvested regarding the cohort, the study design, the surgical technique the nonunion and the complication rate at the longest follow-up. Two groups were built: arthrodeses fixed with screws combined with cancellous autograft (G1) and arthrodeses fixed with screws combined with cancellous autograft and augmented with a lateral fibular onlay (G2). A convenience sample of arthrodeses fixed with cannulated screws and no graft (G3) (with all the relevant raw data) was extracted from a previous study for a further comparison^13^.

### Risk of bias

The modified Coleman Methodology Score (mCMS) was used to assess the quality of studies included, as in previous foot and ankle literature^31^, ranging from 0 to 100. Two investigators performed the mCMS assessment twice (AI and MD), with an interval of 10 days, then discussed the scores when more than a two-point difference was present, until consensus was reached. A score higher than 85 was considered excellent, good from 70 to 84, moderate from 50 to 69 and poor when less than 50.

### Synthesis of results

Baseline data (reported as average value, standard deviation (SD) and range values in the three groups) were tested for normality using a Shapiro–Wilk test and were then compared using the ANOVA (for normally distributed variables) or the Kruskal–Wallis rank sum test (for nonnormally distributed variables). A proportional meta-analysis was run to pool data regarding the nonunion and the complication rate. The ‘metaprop’ command was used to compute 95% confidence intervals using the score statistic and the exact binomial method and incorporate the Freeman-Tukey double arcsine transformation of proportions. Heterogeneity among studies was assessed through the Higgins’ I^2^ statistic and a random-effect model was applied in all cases. A meta regression (metareg module) was used to compare pooled proportions as follows: (1) G1 versus G2; (2) pooled G1 + G2 versus G3. The significance level for the overall estimates of effect was set at *p* < 0.05. All analyses were performed using STATA statistical software package (Version 16.0, StataCorp, 2019).

## Data Availability

Some or all data, models, or code generated or used during the study are available from the corresponding author upon request.
